# Bronchiectasis in renal transplant patients: a cross-sectional study

**DOI:** 10.1186/s40001-024-01701-1

**Published:** 2024-02-13

**Authors:** Pauline Mulette, Jeanne-Marie Perotin, Anaëlle Muggeo, Thomas Guillard, Audrey Brisebarre, Hélène Meyer, Jean Hagenburg, Julien Ancel, Valérian Dormoy, Vincent Vuiblet, Claire Launois, François Lebargy, Gaëtan Deslee, Sandra Dury

**Affiliations:** 1 Department of Respiratory Diseases, Reims University Hospital, Maison Blanche University Hospital, 45, Rue de Cognacq-Jay, 51 092, Reims Cedex, France; 2 Inserm UMR-S 1250, P3Cell, SFR CAP-Santé, University of Reims Champagne-Ardenne, Reims, France; 3 Laboratory of Bacteriology, Virology and Hygiene, Reims University Hospital, Reims, France; 4 Department of Respiratory Diseases, Valenciennes Hospital Center, Valenciennes, France; 5 Department of Nephrology and Renal Transplantation, Reims University Hospital, Reims, France; 6 EA7509 IRMAIC, University of Reims Champagne-Ardenne, Reims, France

**Keywords:** Bronchiectasis, Extended culture, CT scan, Renal transplantation, Quality of life

## Abstract

**Background:**

Bronchiectasis is a chronic airway disease characterized by permanent and irreversible abnormal dilatation of bronchi. Several studies have reported the development of bronchiectasis after renal transplantation (RT), but no prospective study specifically assessed bronchiectasis in this population. This study aimed to compare features of patients with bronchiectasis associated with RT to those with idiopathic bronchiectasis.

**Methods:**

Nineteen patients with bronchiectasis associated with RT (RT-B group) and 23 patients with idiopathic bronchiectasis (IB group) were prospectively included in this monocentric cross-sectional study. All patients underwent clinical, functional, laboratory, and CT scan assessments. Sputum was collected from 25 patients (*n* = 11 with RT-B and *n* = 14 with IB) and airway microbiota was analyzed using an extended microbiological culture.

**Results:**

Dyspnea (≥ 2 on mMRC scale), number of exacerbations, pulmonary function tests, total bronchiectasis score, severity and prognosis scores (FACED and E-FACED), and quality of life scores (SGRQ and MOS SF-36) were similar in the RT-B and IB groups. By contrast, chronic cough was less frequent in the RT-B group than in the IB group (68% vs. 96%, *p* = 0.03). The prevalence and diversity of the airway microbiota in sputum were similar in the two groups.

**Conclusion:**

Clinical, functional, thoracic CT scan, and microbiological characteristics of bronchiectasis are overall similar in patients with IB and RT-B. These results highlight that in RT patients, chronic respiratory symptoms and/or airway infections should lead to consider the diagnosis of bronchiectasis. Further studies are required to better characterize the pathophysiology of RT-B including airway microbiota, its incidence, and impact on therapeutic management.

**Supplementary Information:**

The online version contains supplementary material available at 10.1186/s40001-024-01701-1.

## Background

Non-cystic fibrosis (non-CF) bronchiectasis is a chronic airway disease characterized by permanent and irreversible abnormal dilatation of bronchi [1]. The main causes of bronchiectasis are post-infectious, immunodeficiency, chronic obstructive pulmonary disease, connective tissue disease, ciliary dysfunction, and allergic bronchopulmonary aspergillosis. However, despite extensive etiologic investigations, bronchiectasis remains considered idiopathic in 45% of the cases [2, 3]. The most common symptoms of bronchiectasis are cough, sputum, dyspnea, and fatigue [4] which are associated with impaired quality of life [5–7] and frequent exacerbation [8]. The airways of non-CF bronchiectasis are predisposed to microbial colonization and increased risk of chronic infection [9]. At a stable state, the most common bacteria are *Haemophilus influenzae* and *Pseudomonas aeruginosa* [10–12].

Several studies have previously reported the development of bronchiectasis after renal transplantation (RT) in children and adults [13–16]. Recently, we conducted a multicenter retrospective study describing the clinical, functional, radiological and microbiological characteristics of 46 patients with bronchiectasis revealed after RT [17]. This study identified frequent symptoms of chronic cough and sputum, frequent airway infections with *H. influenzae*, and a mean time of 11 years between RT and the diagnosis of bronchiectasis. The pathophysiology of bronchiectasis associated with RT is not yet elucidated. It may involve hypogammaglobulinemia induced by immunosuppressive drugs, a potential direct effect of mycophenolic acid, and/or predisposing factors associated with the underlying renal disease especially autosomal dominant polycystic kidney disease (ADPKD) [17]. Although RT is the most common form of solid organ transplantation, no prospective study specifically assessed bronchiectasis in this population. Moreover, no study compared the clinical features of bronchiectasis associated with RT (RT-B) to idiopathic bronchiectasis (IB) in terms of respiratory symptoms, pulmonary function, quality of life, and airway microbiota.

This study aimed to compare the clinical features of RT-B patients to those with IB. In addition, we compared the viable airway microbiota at a stable state between RT-B and IB patients.

## Methods

### Study population

This prospective cross-sectional monocenter study was conducted in the Department of Respiratory Diseases at Reims University Hospital (France) from November 2016 to December 2019. Patients were included in the cohort for research and innovation in inflammatory respiratory diseases (Recherche et INNOvation en PAthologie Respiratoire Inflammatoire: RINNOPARI). This study was approved by the ethics committee (Comité de Protection des Personnes—Dijon EST I, No. 2016-A00242-49) and registered in clinicaltrials.gov (NCT02924818). The authorization to access patient data was obtained from the French Advisory Committee for Data Processing in Health Research (CCTIRS, Comité Consultatif sur le Traitement de l’Information en matière de Recherche dans le domaine de la Santé) (no. 13.018) and approved by the national commission for the personal data protection (CNIL, Comité National de l’Informatique et des Libertés) (no. 913412). Data were fully anonymized. Each patient signed written informed consent.

Patients were consecutively included if they were at least 18 years old and matched to one of the two groups: (1) patients with RT and bronchiectasis (RT-B group) or (2) patients without RT and with bronchiectasis considered as idiopathic (IB group). Causative diseases of bronchiectasis including cystic fibrosis, common variable immunodeficiency, allergic bronchopulmonary aspergillosis, asthma, alpha-1-antitrypsin deficiency, chronic obstructive pulmonary disease, rheumatoid arthritis, inflammatory bowel disease, or mycobacterial lung infection sequelae were considered as exclusion criteria [1, 18]. Patients were also excluded if the respiratory disease was not at a stable state defined by the absence of airway infection requiring antibiotics in the last month.

### Data collection

Demographic, clinical, functional (pulmonary function tests, 6-min walking test, arterial blood gas), laboratory, and microbiological data and thoracic computed tomography (CT)-scan results were recorded in a standardized format. Symptoms and quality of life scores were evaluated using four scales: the *Cough And Sputum Assessment Questionnaire (CASA-Q*) [19], the *Hospital Anxiety and Depression Scale (HAD)* [20, 21], the *St George’s Respiratory Questionnaire (SGRQ)* [22, 23], and the *Medical Outcome Study Short Form 36 health survey (MOS SF-36)* [24, 25].

Measurement of full blood count, serum creatinine, level of total immunoglobulins (Ig), and dosage of immunosuppressive drugs for RT-B group were performed.

According to the international consensus, an exacerbation was defined by an impairment of at least 3 or more baseline symptoms (cough, sputum volume or purulence, breathlessness, fatigue, malaise, hemoptysis) for at least 48 h and requiring a change in treatment [26].

The severity and prognosis of bronchiectasis were evaluated according to two multidimensional grading systems: the FACED [27] and the E-FACED score [28].

### CT scans

Each CT scan was reviewed by two pulmonologists (SD, GD) with a final consensus interpretation. All CT scans were performed with the patient in the supine position at end-inspiratory volume using multidetector CT scanners. One- to 5-mm-thick slices at 5- to 10-mm intervals were analyzed from the lung apices to the lung bases. The diagnosis of bronchiectasis was defined according to the Fleischner Society as dilated bronchial lumen relative to the adjacent pulmonary artery, absence of bronchial tapering, and visualization of bronchi within 1 cm of the pleural surface [29]. The extent of bronchiectasis, the thickness of the bronchial wall, and small airways abnormalities were quantified for each lobe (lingula was considered as a separated lobe) according to the *Ooi* score [30].

### Microbiology

Sputum samples were collected after patients rinsed their mouths out with sterile water. If spontaneous sputum was not possible, induced sputum was systematically performed according to international recommendations [31, 32].

Extended culture analysis was performed on the sputum. After liquefaction by *N*-acetylcysteine, serial dilutions (1/1000, 1/10,000, and 1/100,000) were made and cultured in Columbia blood agar, chocolate agar, Schaedler agar, and *Pseudomonas* selective cetrimide agar (Thermo Fisher Scientific, USA), at 37 °C for 48 h for aerobic and 5% CO_2_ cultures and 5 days for anaerobic cultures. All colonies that appeared to be morphologically distinct were quantified as colony-forming unit (CFU) per milliliter and identified by matrix-associated laser desorption ionization-time of flight (MALDI-TOF) mass spectrometry (MALDI Biotyper®, Bruker Daltonics, Bremen, Germany). The α-diversity of the airway microbiota was evaluated with the Shannon index (a marker of intra-individual diversity).

Chronic *P. aeruginosa* infection was defined by the isolation of *P. aeruginosa* in two or more cultures, at least 3 months apart in a consecutive period of 12 months at a stable state [33].

### Statistical analysis

Statistical analysis was performed using SPSS software (version 26). Data are expressed as mean (standard deviation) or median (25th or 75th percentiles) depending on data distribution for quantitative variables and as numbers (%) for qualitative variables. Comparisons were performed using Chi^2^ or fisher’s exact test for qualitative variables and Student’s *t*-test for quantitative variables. A *p* value < 0.05 was considered significant.

## Results

### Patients characteristics

Clinical characteristics of the 42 patients anlayzed are shown in Table 1. Among 44 patients included, two patients were excluded from the IB group because of underlying causes (rheumatoid arthritis *n* = 1, alpha-1 antitrypsin deficiency *n* = 1). Data were analyzed for 19 patients in the RT-B group and 23 patients in the IB group. RT-B and IB groups were similar in terms of age, sex ratio, body mass index (BMI), and smoking history. Dyspnea (≥ 2 on modified Medical Research Council scale (mMRC)), hemoptysis, and number of exacerbations were similar in the RT-B and IB groups. By contrast, the chronic cough was more frequent in the IB group when compared with the RT-B group. The median interval between first RT and first respiratory symptoms and between first RT and diagnosis of bronchiectasis was 11 (1–20) and 12 (2–18) years, respectively. Most RT-B patients (79%) had undergone only one renal transplantation.

**Table 1:** Clinical characteristics

	RT-B *n* = 19	IB *n* = 23	*P* value
Male	12 (63)	11 (48)	0.320
Age, years	66 (57–69)	62 (48–70)	0.147
Smoker (current or former)	12 (63)	9 (39)	0.121
Pack-years	26 (14–30)	10 (7–25)	0.652
Clinical features			
BMI, kg/m^2^	25 (23–30)	23 (21–26)	0.144
Chronic cough	13 (68)	22 (96)	**0.034**
Dyspnea			
mMRC ≥ 2^a^	8 (53)	9 (39)	0.768
Hemoptysis	0 (0)	2 (9)	0.215
Respiratory tract infections			
Exacerbation in the past year	12 (63)	19 (83)	0.637
Number of episodes per patient	1 (0–2)	1 (1–3)	0.551
Hospitalization for exacerbation in the past year	6 (32)	1 (4)	0.189
Cause of end-stage renal disease			
Chronic glomerulonephritis	4 (21)	NA	
Diabetic nephropathy	2 (11)	NA	
Autosomal dominant polycystic kidney disease	6 (32)	NA	
Chronic tubulointerstitial nephritis	2 (11)	NA	
Unknown cause of end-stage renal disease	3 (16)	NA	
Other renal^b^	2 (11)	NA	
Immunosuppressive drugs			
Mycophenolate mofetil	14 (74)	NA	
Cyclosporine	8 (42)	NA	
Tacrolimus	7 (37)	NA	
Azathioprine	2 (11)	NA	
Oral corticosteroids	5 (26)	NA	
Antibiotic therapy in the last 6 months	12 (63)	15 (65)	1.000
Interval between first RT and first symptoms, years^c^	11 (1–20)	NA	
Interval between first RT and diagnosis of bronchiectasis, years^c^	12 (2–18)	NA	

Data are expressed as median (25th or 75th percentiles) and as number (percentage). *p*-value < 0.05 are considered as significant and highlighted in bold

*BMI* body mass index, *IB* patients with idiopathic bronchiectasis, *mMRC* modified Medical Research Council, *NA* not applicable, *RT-B* patients with renal transplantation and bronchiectasis

^a^Missing data for 4 patients in RT-B group

^b^Dysplastic kidney disease (malformation of the kidney)

^c^One patient exhibited bronchiectasis before transplantation

Functional characteristics and radiological data are shown in Table 2. There was no difference between the RT-B and IB groups regarding pulmonary function tests, 6-min walking test and arterial blood gas. The total bronchiectasis score was similar in the RT-B and IB groups except for the middle lobe exhibiting a higher bronchiectasis score in the IB group than in the RT-B group [4 (1–4) vs. 2 (1–3); *p* = 0.038].

**Table 2:** Functional characteristics and CT scan data

	RT-B *n* = 19	IB *n* = 23	*P* value
Pulmonary function tests			
FEV_1_, %	85 ± 25	80 ± 27	0.593
FVC, %	90 ± 21	88 ± 22	0.758
FEV_1_/FVC < 0.70	6 (32)	6 (26)	0.694
RV, %	135 ± 27	138 ± 54	0.794
TLC, %	103 ± 14	103 ± 14	0.949
DLCO, %	59 ± 16	64 ± 19	0.393
6-min walking test (room air)^a^			
SpO_2_ min, %	94 ± 4	93 ± 5	0.900
Distance, m	390 ± 99	459 ± 104	0.067
Arterial blood gas (room air)^b^			
PaO_2_, mmHg	82 ± 15	81 ± 16	0.883
Bronchiectasis score^c,d^			
Total score	16 (6–22)	17 (10–28)	0.169

Data are expressed as mean (± standard deviation), median (25th or 75th percentiles) and number (percentage).

*DLCO* diffusing capacity of the lungs for carbon monoxide, *FEV*_*1*_ forced expiratory volume in 1 s, *FVC* forced vital capacity, *IB* patients with idiopathic bronchiectasis, *PaO*_*2*_ partial pressure of oxygen, *RT-B* patients with renal transplantation and bronchiectasis, *RV* residual volume, *SpO*_*2*_ pulse oxygen saturation, *TLC* total lung volume

^a^Missing data for 7 patients in IB group

^b^Missing data for 7 patients in RT-B group and 5 patients in IB group

^c^Missing data for 2 patients in IB group

^d^According to the Ooi score [30]

Symptoms or prognostic scores and quality of life scales are shown in Table 3. The severity scores (FACED and E-FACED) were similar in the RT-B and IB groups. Regarding the quality of life, the SGRQ total score was similar between the RT-B group and the IB group. There was no significant difference between the RT-B and IB groups in domains of the MOS SF-36 questionnaire. By contrast, cough on the CASA-Q score was more impaired in the IB group compared with the RT-B group.

**Table 3:** Symptoms scores and quality of life scales

	RT-B *n* = 19	IB *n* = 23	*P* value
Severity score			
FACED^a^	2 (1–3)	2 (1–3)	1.000
E-FACED^b^	2 (0–3)	3 (1–4)	0.200
Symptoms score			
CASA-Q			
Cough symptom	75 (50–100)	42 (17–58)	**0.0004**
Cough impact	82 (56–100)	50 (25–81)	**0.019**
Sputum symptom	75 (50–100)	50 (33–67)	**0.008**
Sputum impact	88 (71–100)	75 (38–83)	**0.012**
HAD			
Anxiety (mean)	7 (3–10)	7 (3–10)	0.902
Depression (mean)	6 (2–7)	6 (2–8)	0.805
Quality of life scales			
SGRQ			
Total score	38 (1–58)	38 (22–61)	0.219
MOS SF-36			
Global physical score	36 (24–43)	36 (24–50)	0.745
Global mental score	27 (5–47)	28 (16–44)	0.244

Data are expressed as median (25th or 75th percentiles). *p*-value < 0.05 are considered as significant and highlighted in bold

*IB* patients with idiopathic bronchiectasis, *RT-B* patients with renal transplantation and bronchiectasis

CASA-Q: *Cough And Sputum Assessment Questionnaire* score assessing impact of cough and sputum production on daily activities, ranging from 0 to 100, with the highest score corresponding to a better quality of life; HAD: *Hospital Anxiety and Depression Scale (HAD*: a score between 0 and 7 for anxiety or depression domain meant no trouble, 8–10 suspected troubles, 11–21 confirmed trouble; SGQR: *St George’s respiratory questionnaire (SGRQ)* assessing symptoms and impact on daily activities with a score ranging from 0 to 100, that indicates the maximum impairment of quality of life; MOS SF-36: *Medical Outcome Study Short Form 36 health survey,* a multidimensional generic scale evaluating health status regardless of causal disease, sex, age, and treatment. A Physical Composite Score and a Mental Composite Score was calculated. A score of 100 indicates no impairment of quality of life [24]

^a^FACED score incorporates five variables: forced expiratory volume in 1 s (FEV_1_) % predicted, age, chronic colonization by *P. aeruginosa*, extension of bronchiectasis by radiological assessment, and dyspnea

^b^E-FACED score also includes the occurrence of exacerbations in the previous year

### Laboratory data

The frequency of lymphopenia and hypogammaglobulinemia was not different in the RT-B group compared to the IB group. Dosage of immunosuppressive drugs was within the therapeutic targets in all patients in the RT-B group. As expected, estimated glomerular filtration rate was more impaired in the RT-B group than in the IB group. IgG4 level was lower in the RT-B group compared to the IB group [0.2 (0.0–0.4) vs. 0.7 (0.2–1.1), *p* = 0.032] (Additional file 1: Table S1).

### Microbiological data

**Fig. 1:**
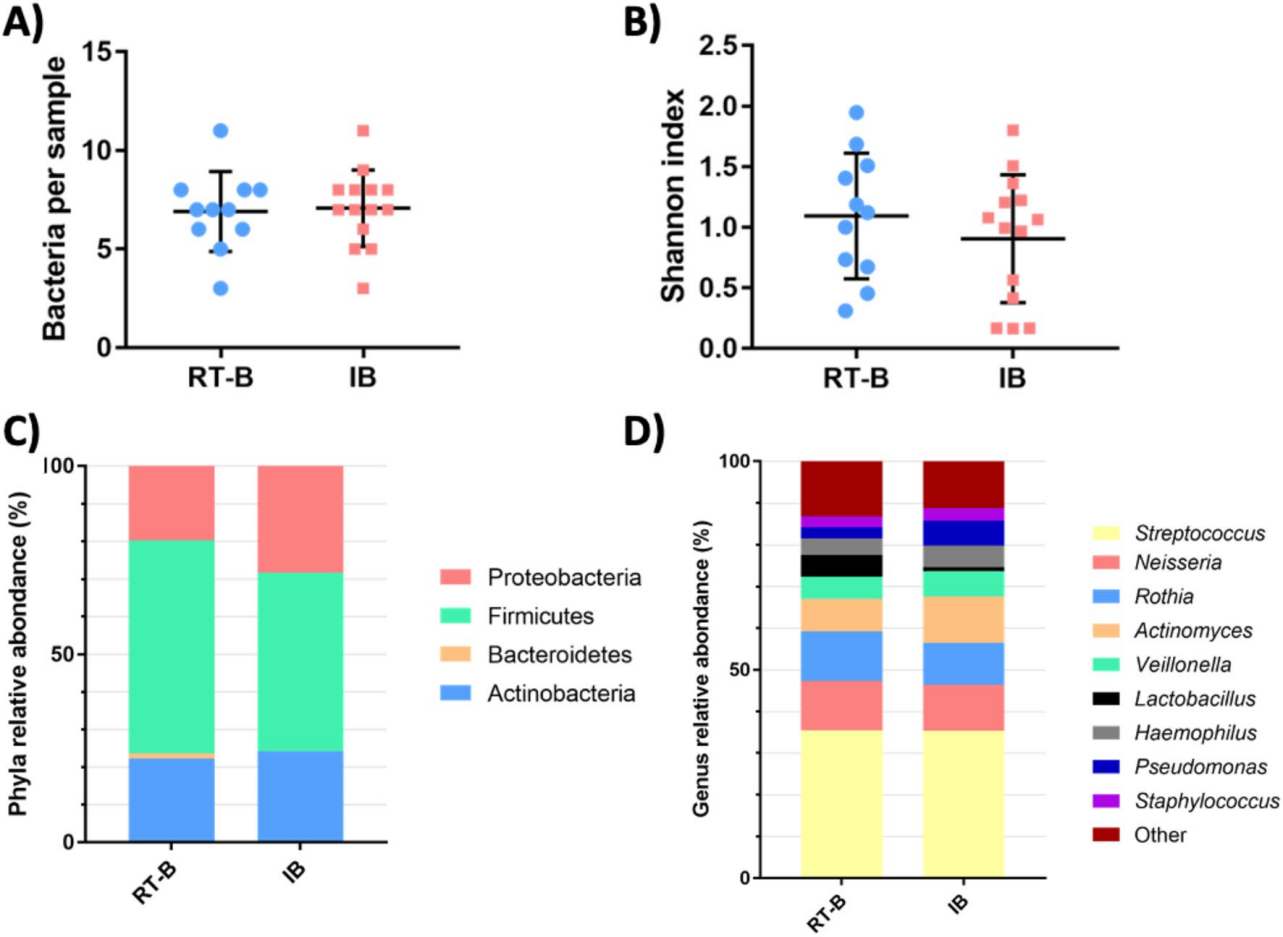
Bacterial diversity of airway microbiota in RT-B and IB patients. **A** Number of bacteria per sample. **B** Alpha diversity (Shannon index). Relative abundance on the phyla level (**C**) and genus level (**D**)

We determined the viable airway microbiota of 25 sputa (11 for RT-B patients and 14 for IB patients). In the RT-B group, we obtained 34 different species with a mean of 6.9 species per sample. In the IB group, we obtained 33 different species with a mean of 7.1 species per sample (Fig. 1A). Assessment of α-diversity revealed no significant differences between the 2 groups (Fig. 1B). A small and non-significant increase of firmicutes and depletion of proteobacteria were observed in the RT-B group (respectively, *p* = 0.23 and *p* = 0.15) (Fig. 1C). The different genera found showed a similar repartition in the two groups, with a predominance of the *Streptococcus*, *Neisseria,* and *Rothia* (Fig. 1D).

**Fig. 2:**
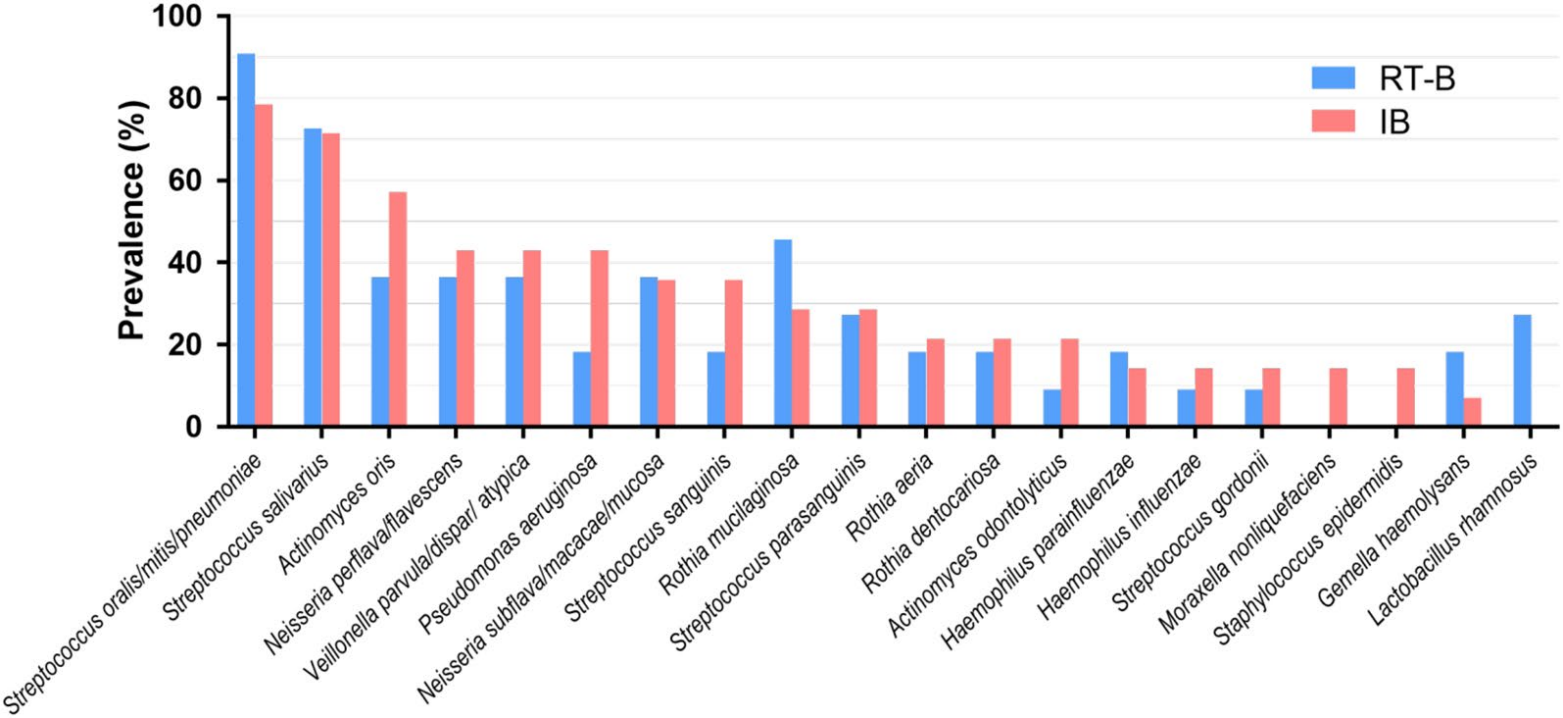
Prevalence of the main bacteria in airway microbiota in RT-B and IB patient sputa. Isolates with less than 10% frequency are not listed. *IB* patients with idiopathic bronchiectasis, *RT-B* patients with renal transplantation and bronchiectasis

We next compared the prevalence of the different species of the microbiota in both RT-B and IB groups (Fig. 2). Two *Streptococci*, *S. oralis/mitis/pneumoniae* and *S. salivarius*, were the most common bacteria, being found in more than 60% of the patients. Although not statistically significant, *Lactobacillus rhamnosus* was more common in RT-B patients than in IB patients (27% in the RT group vs. 0% in the IB group, *p* = 0.072). We also found some pathogenic bacteria at a stable state in this cohort including *P. aeruginosa, H. influenzae,* and *S. aureus* (Additional file 1: Table S2).We observed a trend with less *P. aeruginosa* in the RT-B group than in the IB group (18.2 vs 42.9%, *p* = 0.19), and all patients exhibiting *L. rhamnosus* in the RT-B group did not co-carry *P. aeruginosa.*

Bacterial quantifications ranged from 1 × 10^2^ to 1 × 10^9^, with a median at 1 × 10^5^ and no difference between the two groups of patients. The quantification of the three pathogens *P. aeruginosa*, *H. influenzae,* and *S. aureus* were all higher than this global median, from 1 × 10^6^ to 1 × 10^8^.

## Discussion

This prospective cross-sectional study allowed us to characterize the features of patients with bronchiectasis associated with RT and to compare these features to patients with IB. This study demonstrates that RT-B patients share many clinical features with IB patients including chronic respiratory symptoms, exacerbations, and impaired quality of life. At a stable state, the microbiota of IB and RT-B groups are nearly similar in terms of richness, diversity, and prevalence of different phyla and genera, except for a higher prevalence of *L. rhamnosus* in RT-B patients.

Chronic cough was frequent in RT-B patients (68%) but lower than in IB patients. By contrast, patients with RT-B had similar symptoms of dyspnea and rate of exacerbation per year. Interestingly, the incidence and number of exacerbations in the past year were similar in the RT-B group (63% of patients, median of 1.0 exacerbation per patient) and the IB group (83% of patients, median of 1.0 exacerbation per patient). In a prospective cohort including 608 patients with non-CF bronchiectasis, only 21% of patients had at least one exacerbation in the past year [34], suggesting that our IB group might be more severe due to a selection bias of IB patients followed up in a tertiary university hospital. In both groups, the pulmonary functional impact was limited with mild impairment of forced expiratory volume in 1 s (FEV_1_), consistent with another prospective study including patients with IB showing a mean FEV_1_ of 78% [35]. In our study, the diffusion capacity of carbon monoxide (DLCO) was impaired in 68% (RT-B) and 61% (IB) of the patients, with a mean DLCO between 59 and 64%, respectively. Comparison with literature is limited by few data available regarding DLCO in bronchiectasis. In a study assessing DLCO in non-CF bronchiectasis, 56% of patients had a reduction in DLCO [36]. King et al. reported a mean DLCO value of 88 ± 21%, with a rate of 23% of current or former smokers [37]. The lower DLCO value in our study may be related to a higher rate of current or former smokers in both groups (63% in the RT-B group, 39% in the IB group). Interestingly, the mean DLCO value in RT patients without bronchiectasis was normal in previous studies ranging from 83% [38] to 84% [39]. Regarding CT scan, the bronchiectasis score was similar between the IB and RT-B groups (15 and 20, respectively) assessed by the Ooi et al. score [40].

In our study, FACED and E-FACED scores were similar in RT-B and IB groups, suggesting a similar severity and prognosis despite immunosuppression in the RT group. Of note, there was a trend of lymphopenia in the RT-B group compared to the IB group. The most common causes of mortality in RT patients are cardiovascular disease, followed by cancer and infections (mainly urinary tract and lung infections) [41, 42]. In RT patients, the incidence of pulmonary infection was 8.8% [43]. However, no information regarding thoracic CT scan characteristics including the presence of bronchiectasis was available in these studies. The quality of life in RT patients is associated with general health assessment, physical functioning, pain, sleep quality, occupational status, vitality, social activity, staff support, and quality of care [44]. Mean values of MOS SF-36 global physical and mental scores in RT-B patients seemed more impaired than previously reported in RT patients [45]. However, quality of life was overall similar between RT-B and IB in our study.

Few microbiological data on RT-B patients are available and are limited to case reports [14, 15] and one retrospective study [17]. In this study, we described for the first time the airway viable microbiota of patients with RT-B at a clinically stable state using an extended-quantitative bacterial culture of sputum samples with detection and identification of isolated bacteria. We found that the microbiota was globally similar between RT-B and IB groups. We described the same richness, diversity, and prevalence of the different phyla and genera, with a predominance of the *Streptococci*, *Neisseria*, *Rothia,* and *Veillonella*, as usually described in the airway microbiota in bronchiectasis [9, 45, 46]. Some pathogenic bacteria were detected as part of the microbiota such as *P. aeruginosa*, *H. influenzae,* and *S. aureus*, as reported in other studies from non-CF bronchiectasis patients in Europe [34, 46–48].

The prevalence of all the different bacteria was similar in the 2 groups. Although not statistically significant, probably due to the low number of samples, we found that *L. rhamnosus* was more common in RT-B patients compared to IB patients (27% vs. 0%) and that *P. aeruginosa* was less common in the RT-B group than in the IB group (18% vs. 43%)*.* We also noticed that the patients with *L. rhamnosus* did not co-carry *P. aeruginosa*. This inverse correlation may suggest a protective effect of *L. rhamnosus*, a known probiotic agent, on the carriage of *P. aeruginosa* in airway microbiota. Indeed, there is a growing interest in the potential use of *Lactobacilli* probiotics, notably *L. rhamnosus*, as clinical studies showed the prevention of pneumonia after oral or respiratory administration [49–52]. Many studies also described the abilities of *Lactobacilli* to specifically protect against *P. aeruginosa* infections in murine models of pneumonia [53–55]. Further studies, with an increased number of patients, are needed to confirm this potential protective effect of *Lactobacilli* in bronchiectasis.

There are several limitations to our study. First, it was a monocenter study with a small sample size with a potential selection of more severe IB patients. Second, the small number of patients did not allow us to investigate the potential role of ADPKD as a risk factor of bronchiectasis as previously suggested [56, 57]. However, in our study, the main underlying renal disease in RT-B patients was also ADPKD (*n* = 6, 32%). Third, this study was conducted at a stable state. Some patients were therefore not able to produce sputum and were not included in the microbiological analyzes. Fourth, we did not use the Bronchiectasis Health Questionnaire, which has been developed and validated specifically for patients with bronchiectasis, but was not available when our study started [58]. Finally, the cross-sectional design does not provide information regarding the evolution of the clinical, functional, CT scan, and microbiological features of RT-B over time which would require longitudinal studies with long-term follow-up.

## Conclusion

This cross-sectional study showed that RT-B patients share many clinical features with IB patients including chronic respiratory symptoms, exacerbations, pulmonary function, and quality of life impairment. At a stable state, the microbiota of IB and RT-B groups are nearly similar in terms of richness, diversity, and prevalence of different phyla and genera. These results highlight that bronchiectasis should be considered in RT patients exhibiting chronic respiratory symptoms and/or exacerbation. We hope these results will stimulate to conduct further larger longitudinal studies to better characterize the mechanisms of RT-B including monitoring of airway microbiota in RT, its incidence, and potential impact on therapeutic management.

### Supplementary Information

**Additional file 1: Table S1.** Laboratory data.

**Additional file 2: Table S2.** Prevalence and quantification of the bacteria in airway microbiota in RT-B and IB patients.

## Data Availability

The datasets used and/or analyzed during the current study are available from the corresponding author on reasonable request.

## Abbreviations

ADPKD: Autosomal dominant polycystic kidney disease
BMI: Body mass index
CASA-Q: Cough and Sputum Assessment Questionnaire
CF: Cystic fibrosis
CFU: Colony-forming unit
CT: Computed tomography
DLCO: Diffusing capacity of the lungs for carbon monoxide
FEV_1_: Forced expiratory volume in 1 s
FVC: Forced vital capacity
HAD: Hospital Anxiety and Depression Scale
IB: Idiopathic bronchiectasis
Ig: Immunoglobulin
MALDI-TOF: Matrix-associated laser desorption ionization-time of flight
mMRC: Modified Medical Research Council
RT: Renal transplantation
RT-B: Renal transplantation and bronchiectasis.
MOS SF-36: Medical Outcome Study Short Form 36 health survey
PaO_2_: Partial pressure of oxygen
SGRQ: St George’s Respiratory Questionnaire
SpO_2_: Pulse oxygen saturation
TLC: Total lung volume
