# Supplementary material for: Bronchiectasis in renal transplant patients: a cross-sectional study
Source: Eur J Med Res. 2024 Feb 13;29:120. doi: 10.1186/s40001-024-01701-1 (PMC10863148; doi:10.1186/s40001-024-01701-1)
Supplement: Supplementary file 1 — Additional file 1: Table S1. Laboratory data. [file 40001_2024_1701_MOESM1_ESM.pdf]

Table S1: Laboratory data

	RT-B n=19	IB n=23	*P value*
Serum creatinine, µmol/L	145 (105-212)	69 (62-86)	**0.001**
#eGFR, ml/min/1,73m^2	40 (28-66)	90 (80-109)	**<0.0001**
Lymphocytes, /mm^3			
Lymphopenia	9 (47)	6 (26)	0.064
T lymphocytes (CD3+)	891 (612-1387)	1022 (750-1152)	0.799
CD4+ T lymphocytes	435 (337-568)	613 (412-893)	0.379
CD8+ T lymphocytes	385 (191-471)	391 (198-497)	0.920
B lymphocytes (CD19+)	97 (44-177)	65 (33-168)	0.709
Immunoglobulin assay, g/L			
IgG	11.1 (9.6-11.5)	12.0 (10.3-13.8)	0.093
IgG1	7.7 (5.1-8.3)	7.6 (6.2-9.0)	0.387
IgG2	2.7 (2.2-3.3)	3.1 (2.6-4.5)	0.176
IgG3	0.5 (0.3- 1.1)	0.5 (0.4-0.9)	0.551
IgG4	0.2 (0.0-0.4)	0.7 (0.2-1.1)	**0.032**
IgA	2.7 (1.5-3.7)	3.0 (2.0-4.0)	0.836
IgM	0.7 (0.4-1.0)	1.1 (0.6-1.4)	0.336
Hypogammaglobulinemia	1 (5)	0 (0)	0.276
Plasma concentration of immunosuppressive drugs			
Mycophenolate mofetil, mg.h/L	3 (3-5)	NA	
Cyclosporine, µg/L	90 (78-97)	NA	
Tacrolimus, ng/mL	6 (5-8)	NA	

Data are expressed as median (25th or 75th percentiles) and as number (percentage).
eGFR : estimated glomerular filtration rate, IB: patients with idiopathic bronchiectasis, Ig: immunoglobulin, RT-B: patients with renal transplantation and bronchiectasis,.
According to MDRD formula
