# Supplementary material for: Bronchiectasis in renal transplant patients: a cross-sectional study
Source: Eur J Med Res. 2024 Feb 13;29:120. doi: 10.1186/s40001-024-01701-1 (PMC10863148; doi:10.1186/s40001-024-01701-1)
Supplement: Supplementary file 2 — Additional file 2: Table S2. Prevalence and quantification of the bacteria in airway microbiota in RT-B and IB patients. [file 40001_2024_1701_MOESM2_ESM.pdf]

Table S2: Prevalence and quantification of the bacteria in airway microbiota in RT-B and IB patient

RT-B	n = 11	%	Quanti-fication (mediane)	IB	n = 14	%	Quanti-fication (mediane)
Streptococcus oralis/mitis/pneumoniae	10	90,9	1,0E+07	*Streptococcus oralis/mitis/pneumoniae*	11	78,6	1,0E+06
Streptococcus salivarius	8	72,7	5,5E+05	*Streptococcus salivarius*	10	71,4	1,0E+06
Rothia mucilaginosa	5	45,5	1,0E+05	*Actinomyces oris*	8	57,1	5,5E+04
Actinomyces oris	4	36,4	5,5E+04	*Neisseria perflava/flavescens*	6	42,9	5,5E+05
Neisseria perflava/flavescens	4	36,4	5,5E+05	*Pseudomonas aeruginosa*	6	42,9	1,0E+06
Neisseria subflava/macacae/mucosa	4	36,4	1,0E+06	*Veillonella parvula/dispar/ atypica*	6	42,9	5,5E+05
Veillonella parvula/dispar/ atypica	4	36,4	5,5E+04	*Neisseria subflava/macacae/mucosa*	5	35,7	1,0E+05
Lactobacillus rhamnosus	3	27,3	1,0E+05	*Streptococcus sanguinis*	5	35,7	1,0E+06
Streptococcus parasanguinis	3	27,3	1,0E+06	*Rothia mucilaginosa*	4	28,6	5,5E+05
Gemella haemolysans	2	18,2	7,5E+04	*Streptococcus parasanguinis*	4	28,6	1,0E+05
Haemophilus parainfluenzae	2	18,2	5,1E+05	*Actinomyces odontolyticus*	3	21,4	1,0E+04
Pseudomonas aeruginosa	2	18,2	3,0E+07	*Rothia aeria*	3	21,4	1,0E+05
Rothia aeria	2	18,2	5,5E+06	*Rothia dentocariosa*	3	21,4	1,0E+07
Rothia dentocariosa	2	18,2	5,5E+06	*Haemophilus influenzae*	2	14,3	5,1E+08
Streptococcus sanguinis	2	18,2	5,5E+06	*Haemophilus parainfluenzae*	2	14,3	5,1E+06
Actinomyces naeslundii	1	9,1	1,0E+05	*Moraxella nonliquefaciens*	2	14,3	5,5E+06
Actinomyces odontolyticus	1	9,1	1,0E+07	*Staphylococcus epidermidis*	2	14,3	5,1E+05
Bacillus lichenformis	1	9,1	1,0E+05	*Streptococcus gordonii*	2	14,3	5,0E+06
Branhamella catarrhalis	1	9,1	1,0E+07	*Abiotrophia defectiva*	1	7,1	1,0E+06
Capnocytophaga sputigena	1	9,1	1,0E+04	*Achromobacter xylosoxidans*	1	7,1	1,0E+05
Enteroccocus faecalis	1	9,1	5,0E+04	*Acinetobacter lwoffii*	1	7,1	1,0E+04
Granulicatella adiacens	1	9,1	1,0E+06	*Alcaligenes feacalis*	1	7,1	1,0E+03
Haemophilus influenzae	1	9,1	1,0E+06	*Bifidobacterium dentium*	1	7,1	1,0E+03
Lactobacillus gasseri	1	9,1	1,0E+05	*Corynebacterium propinquum*	1	7,1	1,0E+04
Micrococcus luteus	1	9,1	1,0E+04	*Gemella haemolysans*	1	7,1	1,0E+02
Neisseria elongata	1	9,1	1,0E+05	*Haemophilus haemolyticus*	1	7,1	1,0E+04
Parvimonas micra	1	9,1	1,0E+07	*Lactobacillus fermentum*	1	7,1	1,0E+04
Propionibacterium acnes	1	9,1	1,0E+05	*Micrococcus luteus*	1	7,1	1,0E+04
Staphylococcus aureus	1	9,1	1,0E+07	*Morganella morganii*	1	7,1	1,0E+05
Staphylococcus haemolyticus	1	9,1	1,0E+03	*Staphylococcus hominis*	1	7,1	1,0E+04
Streptococcus anginosus	1	9,1	1,0E+04	*Streptococcus anginosus*	1	7,1	1,0E+08
Streptococcus cristatus	1	9,1	1,0E+07	*Streptococcus constellatus*	1	7,1	1,0E+04
Streptococcus gordonii	1	9,1	5,0E+04	*Streptococcus cristatus*	1	7,1	1,0E+04
Streptococcus mutans	1	9,1	1,0E+07				
